# Profiling and functional analysis of circular RNAs in acute promyelocytic leukemia and their dynamic regulation during all-*trans* retinoic acid treatment

**DOI:** 10.1038/s41419-018-0699-2

**Published:** 2018-05-29

**Authors:** Shufen Li, Yunlin Ma, Yun Tan, Xuefei Ma, Ming Zhao, Bing Chen, Rongsheng Zhang, Zhu Chen, Kankan Wang

**Affiliations:** 1 State Key Laboratory of Medical Genomics, Ruijin Hospital, Shanghai Jiao Tong University School of Medicine, Shanghai, 200025 China; 2 Sino-French Research Center for Life Sciences and Genomics, Ruijin Hospital, Shanghai Jiao Tong University School of Medicine, Shanghai, China

## Abstract

Circular RNAs (circRNAs) are a novel class of powerful regulators in gene expression and participate in the pathogenesis of many diseases, including cancer. However, little is known about the roles of circRNAs in the development and treatment of acute promyelocytic leukemia (APL). Here we report the expression profiling and function of circRNAs in APL, including their dynamic regulation during all-*trans* retinoic acid (ATRA)-induced differentiation. We performed two independent ribosomal RNA-minus RNA-sequencing (Ribo-minus RNA-seq) experiments with and without RNase R treatment on APL patient-derived NB4 cells and identified a total of 4313 circRNAs, including 1098 newly identified circRNAs. Detailed analysis showed that circRNAs expressed in APL cells were mostly exon-derived, not by-products during splicing, and could be distinguished from hematopoietic stem cells, neutrophils and lymphocytes. The true presence and stability of circRNAs were verified both in NB4 cells and primary APL patient samples. Moreover, we conducted a time-series analysis of circRNAs on ATRA-treated NB4 cells and uncovered 508 circRNAs with dynamic expression during ATRA treatment, including 246 upregulated and 262 downregulated. Further evidence demonstrated that the majority of circRNAs were regulated independently of their host linear mRNAs. Detailed functional experiments demonstrated that circ-HIPK2, one of the differentially expressed circRNAs, significantly influenced ATRA-induced differentiation of APL cells. Further mechanistic studies revealed that circ-HIPK2 was located in cytoplasm and served as a sponge for differentiation-associated miR-124-3p. Finally, circ-HIPK2 expression in APL patients was significantly lower than that in normal peripheral mononuclear cells and other subtypes of AML, indicating its potential role as an APL biomarker. Our study indicates the biological functions of circRNAs in the development and treatment of APL, and provides a comprehensive circRNA resource for future studies.

## Introduction

Circular RNAs (circRNAs) that are widely expressed in diverse cell types and species have recently emerged as a novel assortment of regulatory non-coding RNAs in gene expression^1,2^. Accumulating evidence indicates that gene expression is a complex process containing multiple layers of regulation and highlights that circRNA regulation is a critical step at the transcriptional and post-transcriptional levels^3,4^. High-throughput RNA-sequencing (RNA-seq) has dramatically accelerated the discovery of circRNAs, allowing us to more accurately investigate circRNAs at the genome-wide scale^2,5,6^. Profiling of circRNA expression has revealed that circRNAs are highly abundant in number^7^, evolutionarily conserved across species^2,8^, and expressed in a tissue-specific or cell type-specific manner^5,9^. These unique features indicate that circRNAs may have a biological function in various cellular processes.

Although the molecular mechanisms of circRNAs are poorly understood, they indeed have been implicated as regulators of diverse cellular processes, including proliferation^10^, differentiation^8^, signaling^11^, and aging^12,13^. Consequently, the aberrant circRNA expression has been indicated to contribute to pathological processes, especially cancer^14,15^. Studies have shown that circRNA deregulation occurs in cancer samples, compared to adjacent non-cancerous tissues^16–18^. In addition, several particular circRNAs have been reported to contribute to the abnormal proliferation and growth of cancer cells, such as circ-TCF25 in bladder carcinoma^19^, circ-PVT1 in gastric cancer^10^, and circ-001569 in colorectal cancer^20^. More importantly, circRNAs can be abundantly detected in peripheral blood, showing the potential of circRNAs as cancer biomarkers^10,21–23^. Hence, the identification of cancer-associated circRNAs and the investigation of their action mechanisms can provide critical insights into the biology of cancer development and progression.

Transcriptional deregulation due to oncogenic fusion proteins derived from chromosomal translocations is a hallmark of the pathogenesis of leukemia^24^. In acute promyelocytic leukemia (APL), the PML/RARα (promyelocytic leukemia-retinoic acid receptor α) fusion protein that is generated by the t(15;17) translocation induces oncogenic transcriptional programs, thus contributing to malignant transformation and a differentiation block at the promyelocytic stage^25–27^. Furthermore, all-*trans* retinoic acid (ATRA) is able to convert the oncogenic transcriptional programs initiated by PML/RARα, thereby resulting in the complete remission in 90% of APL patients^27,28^. In addition to protein-coding genes, a number of non-coding RNAs, such as microRNAs^29,30^ and long non-coding RNAs^31–33^, have been reported to contribute to the transcriptional deregulation of APL as well. The remarkable features of circRNAs, as a newly identified class of regulators in gene expression, prompt the investigation of their physiological significance and in particular the putative roles in the pathogenesis and treatment of APL. Actually, a specific circRNA generated from the PML/RARα fusion site was recently reported to promote APL progression^34^. However, the potential roles of circRNAs in the development and treatment of APL remain largely unknown.

In this study, we aimed to investigate the expression profiling and function of circRNAs in APL, including their dynamic regulation during ATRA-induced differentiation. We performed two independent ribosomal RNA-minus RNA-sequencing (Ribo-minus RNA-seq) experiments, one with and one without RNase R treatment, to identify circRNAs in APL cells before and 24 and 48 h after ATRA treatment. A total of 4313 high confidence circRNAs were repeatedly detected by both experiments. Among these circRNAs, we uncovered 508 differentially expressed circRNAs (246 upregulated and 262 downregulated) upon ATRA treatment and showed that the regulation of circRNAs was independent of their host linear mRNAs, indicating the independent function of circRNAs in ATRA-induced differentiation of APL cells. Furthermore, we found that one of the circRNAs, circ-HIPK2, significantly influenced ATRA-induced differentiation of NB4 cells, indicating that circ-HIPK2 was indispensable for the differentiation of APL. Further investigation of functional mechanisms of circ-HIPK2 showed that circ-HIPK2 could serve as a sponge for microRNAs, especially for miR-124-3p. Finally, the specifically low expression of circ-HIPK2 in APL samples, compared with normal blood samples and other subtypes of AML samples, suggested the putative role of circ-HIPK2 as an APL biomarker. Our findings provide insights on circRNA expression and function in the development and treatment of APL.

## Results

### Overview of circRNA profiles used in this study

To explore the molecular regulation of circRNAs in APL cells and the differentially regulated circRNAs during ATRA treatment, we performed two independent Ribo-minus RNA-seq, one with and one without RNase R digestion, to acquire circRNA profiles in an APL patient-derived cell line NB4 before and 24 and 48 h after ATRA treatment. To identify circRNAs that are specifically expressed in APL cells, we also retrieved public available Ribo-minus RNA-seq data sets from three leukocyte types: hematopoietic stem cells (CD34^+^), naive B cells (CD19^+^) and neutrophils. The circRNA profiles used in this study can be found in Supplementary Table S1.

### Characteristics of circRNAs in APL cells

On the basis of the two independent Ribo-minus RNA-seq, we identified 4313 high-confidence circRNAs that were repeatedly detected in at least one of the three time-points by both experiments (details in Materials and Methods, Supplementary Table S2). Approximately 55–60% of the circRNAs identified in RNase R-untreated RNA-seq data were also detected in the RNase R-treated RNA-seq data, which is consistent with the previous observation achieved between RNase R-untreated and -treated RNA-seq^6,35^. The comparison of these circRNAs with the circBase database (http://circbase.org/) showed that there were 3215 (74.5%) annotated circRNAs and 1098 (25.5%) newly identified circRNAs. The large number of circRNAs identified in NB4 cells supported the previous finding that circRNAs may act as one of the major RNA families.

**Fig. 1:**
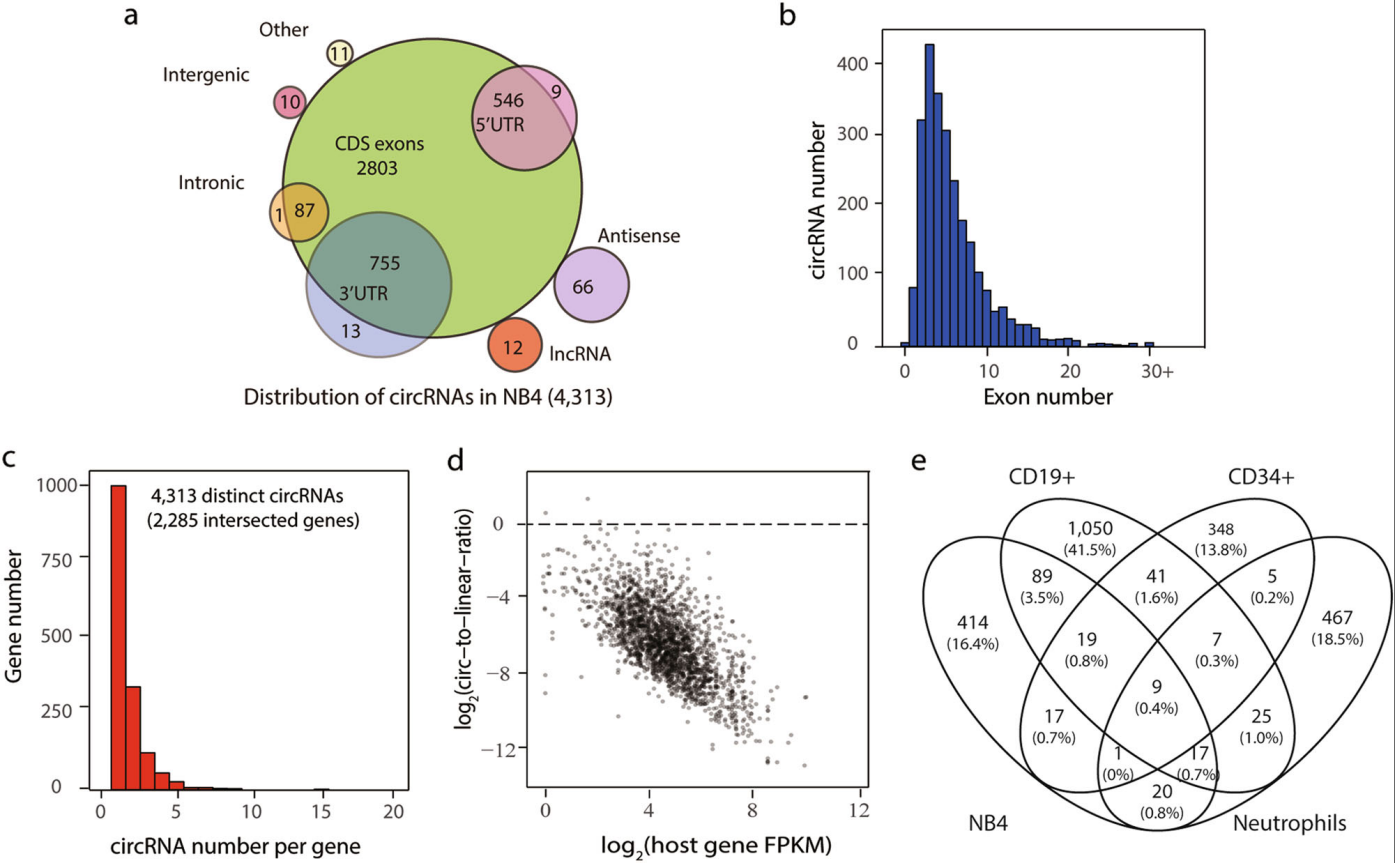
Mapping and genomic characteristics of circRNAs in APL-derived NB4 cells. **a** Genomic distribution of circRNAs identified in NB4 cells. **b** The exon number distribution for exon-derived circRNAs. **c** Distribution of the circRNA number per gene in NB4 cells. **d** The correlation between the ratio of circRNA to linear RNA and the expression of the host gene. **e** Venn diagram showing the overlapped circRNAs among NB4, CD34^+^ cells, CD19^+^ cells and neutrophils

To determine the genomic distribution of circRNAs, we mapped the relative position of circRNAs to the annotated genome in the Ensembl release 75 (http://www.ensembl.org/). As illustrated in Fig. 1a, the majority of the circRNAs (95.7%) were derived from the annotated exon regions, including 5′-UTR, CDS and 3′-UTR. Only a small fraction (4.3%) of circRNAs were located in long non-coding RNAs, antisense regions of known genes and other unannotated regions. Regarding the exon-derived circRNAs, the number of exons within circRNAs showed great diversity (from 1 to more than 30) and most exon-derived circRNAs consisted of two or three exons (Fig. 1b), in accordance with the previous work^36^. Interestingly, we also found that the circRNA-producing genes were capable of generating several different circRNAs. As illustrated in Fig. 1c, 4313 high confidence circRNAs detected in NB4 cells were intersected with 2285 annotated Ensembl genes. Among these circRNA-producing genes, ~57.8% (1320) generated only a single circRNA whereas 42.2% (965) produced more than one circRNA.

We next investigated whether circRNAs identified in NB4 cells were by-products during splicing. The correlation of the ratio of the circular transcript to the linear one with the overall mRNA expression of the host gene was examined. If circRNAs were by-products, the proportion of circRNAs would be almost unchanged with the increment of the expression of host genes. As shown in Fig. 1d, an inverse correlation was observed, suggesting that circRNAs expressed in APL cells were not by-products, consistent with the previous finding in neuron cells^8^.

It has been reported that circRNAs are expressed in a cell type-specific and tissue-specific manner^5,9^. To identify APL-specific circRNAs, we compared the circRNA profile of NB4 cells with those of additional three leukocytes, i.e., CD34^+^ hematopoietic stem cells, CD19^+^ B lymphocytes and neutrophils^5^ (Supplementary Table S3). We compared circRNA expression using the same read length from all the data sets (details in Materials and Methods) and found that 70.6% (414/586) of the circRNAs expressed in NB4 cells were cell type-specific (Fig. 1e).

### Validation of circRNAs in both NB4 cells and APL patient samples

**Fig. 2:**
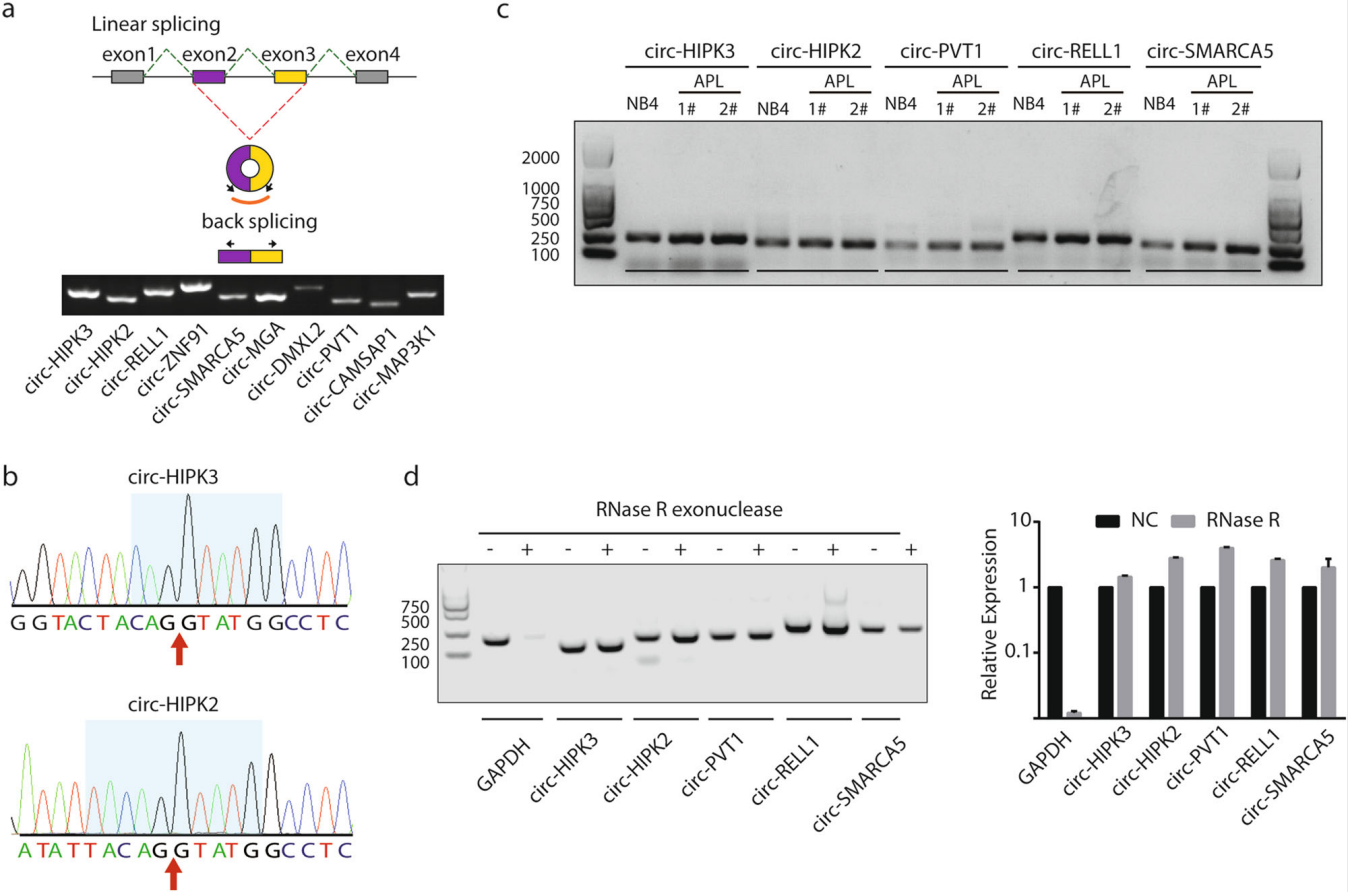
Validation of circRNAs in both NB4 cells and APL patient samples. **a** Validation of circRNAs in NB4 cells by RT-PCR. The upper panel shows the schematic diagram of the divergent primers for RT-PCR. The PCR results of 10 selected circRNAs matched the expected size of circular junctions (the lower panel). **b** Confirmation of circRNAs by sequencing. PCR products of the above 10 circRNAs were sequenced to confirm the back-spliced junction sequence and sites of circRNAs. The back-spliced junction sequence is covered by a blue background and the junction site of each circRNA is indicated by a red arrow. Data of 2 selected circRNAs are shown in this figure and data of the remaining 8 circRNAs can be found in Supplementary Fig. S1. **c** Validation of circRNAs in NB4 cell and bone marrows of two APL patient samples. **d** Validation of circRNAs by RNase R treatment. The selected circRNAs were significantly resistant to RNase R treatment as compared to the linear RNA control GAPDH. Error bars represent means ± s. e. m. of three experiments. The negative control was the linear GAPDH gene. ***P < 0.001

To validate the reliability of circRNAs detected in NB4 cells, 10 circRNAs with different expression levels were randomly selected from our circRNA catalog. According to the junction sequence of each circRNA, we designed special divergent primers for each circRNA (Fig. 2a, the upper panel) and conducted reverse transcription-PCR (RT-PCR) in NB4 cells. The PCR products of all 10 selected circRNAs matched the expected sizes of circular junctions (Fig. 2a, the lower panel), and the junction site of each circRNA was verified by Sanger sequencing (Fig. 2b and Supplementary Fig. S1). We further selected 5 circRNAs from these confirmed circRNAs to verify their expression in two primary APL patient samples. As shown in Fig. 2c, the circRNAs truly existed in both NB4 cells and primary APL patient samples. As circRNAs cannot be degraded by RNase R^2,6^, we thus treated total RNA of NB4 cells with RNase R. After RNase R treatment, we indeed found that circRNAs were significantly enriched while the abundance of the control linear GAPDH was remarkably decreased, confirmed by both RT-PCR and quantitative reverse transcription-PCR (qRT-PCR) (Fig. 2d).

### Identification of differentially expressed circRNAs upon ATRA treatment of APL cells

**Fig. 3:**
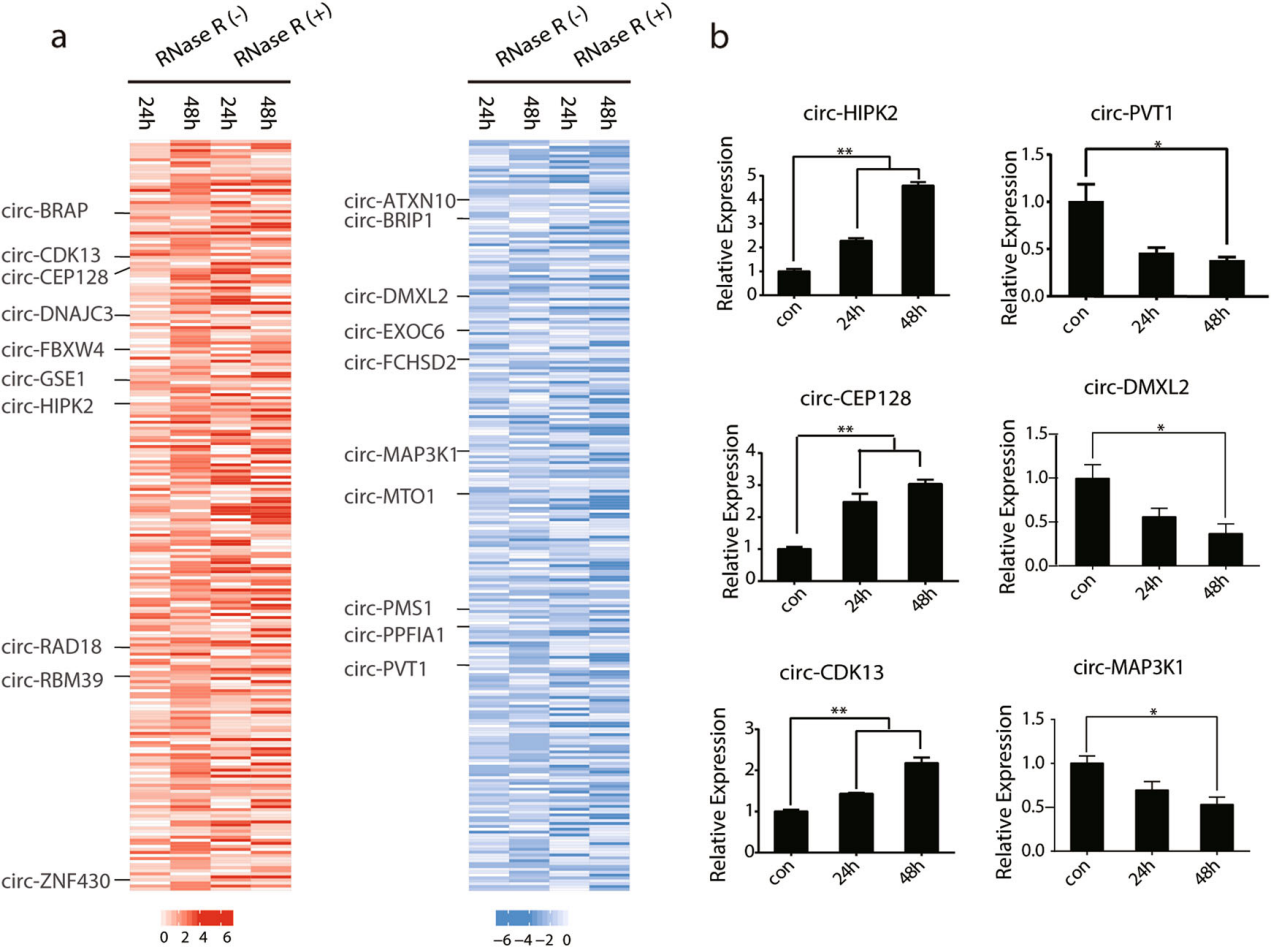
CircRNAs were differentially regulated during ATRA treatment in NB4 cells. **a** Identification of constantly upregulated or downregulated circRNAs upon ATRA treatment for 24 and 48 h, both in the RNase R-untreated and -treated RNA-seq data. 10 upregulated circRNAs and 10 downregulated circRNAs are indicated in the heatmap. **b** Validation of differentially expressed circRNAs upon ATRA treatment. Expression changes of 6 selected circRNAs differentially regulated after ATRA treatment. The data are shown with means ± s. e. m. of three experiments. *P < 0.05, **P < 0.01

APL is characterized by a unique clinical response to a differentiation-inducing agent, ATRA^27,28^. Approximately 90% of APL patients achieve complete clinical remission with ATRA treatment^27^. Much effort has been made to learn about differentially expressed genes upon ATRA treatment of APL cells, but little is known about the differentially expressed circRNAs during this process. To answer this question, we first analyzed the circRNAs that were differentially regulated with ATRA treatment in both RNase R-untreated and RNase R-treated RNA-seq data sets (Supplementary Fig. S2). Among these circRNAs, 246 circRNAs were constantly upregulated and 262 were constantly downregulated (Fig. 3a and Supplementary Table S4). We selected and indicated 10 upregulated and 10 downregulated circRNAs during ATRA-induced APL differentiation in the heatmap of Fig. 3a. These circRNAs or their host genes were reported to be involved in the pathogenesis or treatment process of tumorigenesis. For example, a recent study showed that circ-PVT1 can promote cell proliferation in gastric cancer by serving as an efficient sponge for the miR-125 family^10^. Interestingly, we also found that some of the differentially expressed circRNAs (e.g., circ-HIPK2, circ-DNAJC3, circ-CEP128, circ-FCHSD2) were derived from protein-coding genes with critical roles in hematopoiesis and differentiation^37–41^, also implicating the potential roles of circRNAs in the development and treatment of APL.

From the constantly regulated circRNAs, 3 upregulated and 3 downregulated circRNAs were selected to validate their expression during ATRA-induced differentiation of NB4 cells. As shown in Fig. 3b, the results obtained by qRT-PCR were indeed consistent with those from RNA-seq. Together, these results indicated that circRNAs showed a dynamic regulation pattern during ATRA-induced differentiation of APL cells.

### Mutual independence between most regulated circRNAs and their host linear transcripts during ATRA treatment

**Fig. 4:**
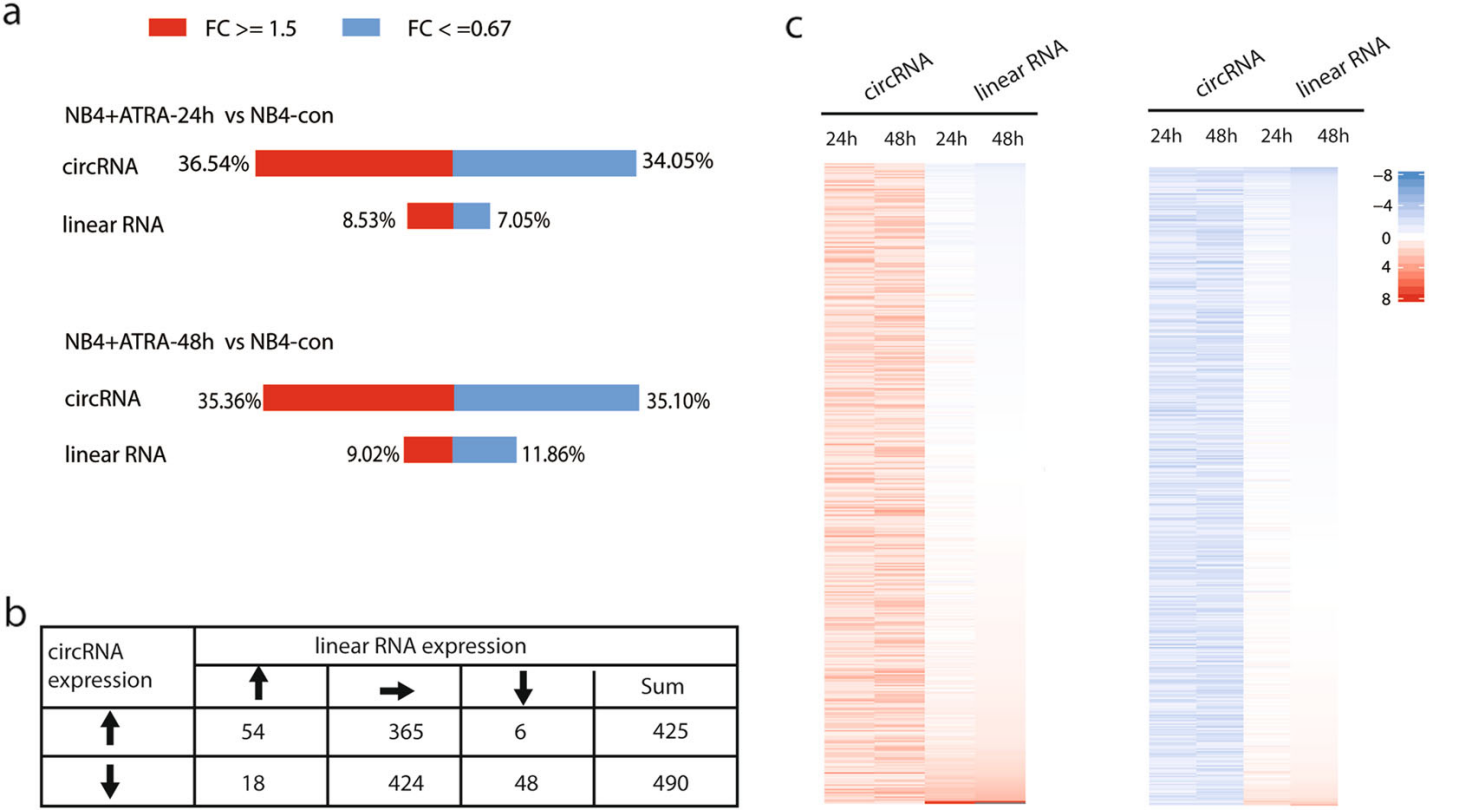
Most circRNAs were independently regulated of their host linear RNAs during ATRA-induced differentiation of NB4 cells. **a** Barplots showing the percentage of differentially expressed circRNAs and corresponding linear RNAs at the two time-points of ATRA treatment. **b** Relationship between differentially expressed circRNAs and their host linear RNAs. Up arrow: upregulated; down arrow: downregulated; right arrow: unchanged; sum: summary. **c** Heatmap showing the expression changes of differentially regulated circRNAs and their host linear RNAs upon ATRA treatment

Given that most circRNAs are derived from protein-coding genes and circRNAs are more stable than linear mRNAs^6^, we next investigated the relationship between differentially expressed circRNAs and their host linear mRNAs. For those circRNAs generated from the same gene, only the circRNA that had the highest expression was calculated. First, a side-by-side comparison between these two classes of RNA transcripts revealed that the number of differentially expressed circRNAs was much more than that of differentially expressed linear RNAs (Fig. 4a). Second, we found that the majority of differentially expressed circRNAs (789/915, 86.2%) was regulated but without the change of their host linear RNAs (Fig. 4b), while only 11.1% (102/915) were regulated simultaneously with their host linear RNAs. Interestingly, there were 24 circRNAs even showing a reciprocal expression pattern with their host linear RNAs. A global view of all differentially expressed circRNAs clearly supported the above finding (Fig. 4c). Taken together, these results suggested that the expression change of the majority of differentially expressed circRNAs might differ from their host linear RNAs, indicating the independent function of circRNAs in ATRA-induced differentiation of APL cells.

### The requirement of circ-HIPK2 in the differentiation of APL cells

**Fig. 5:**
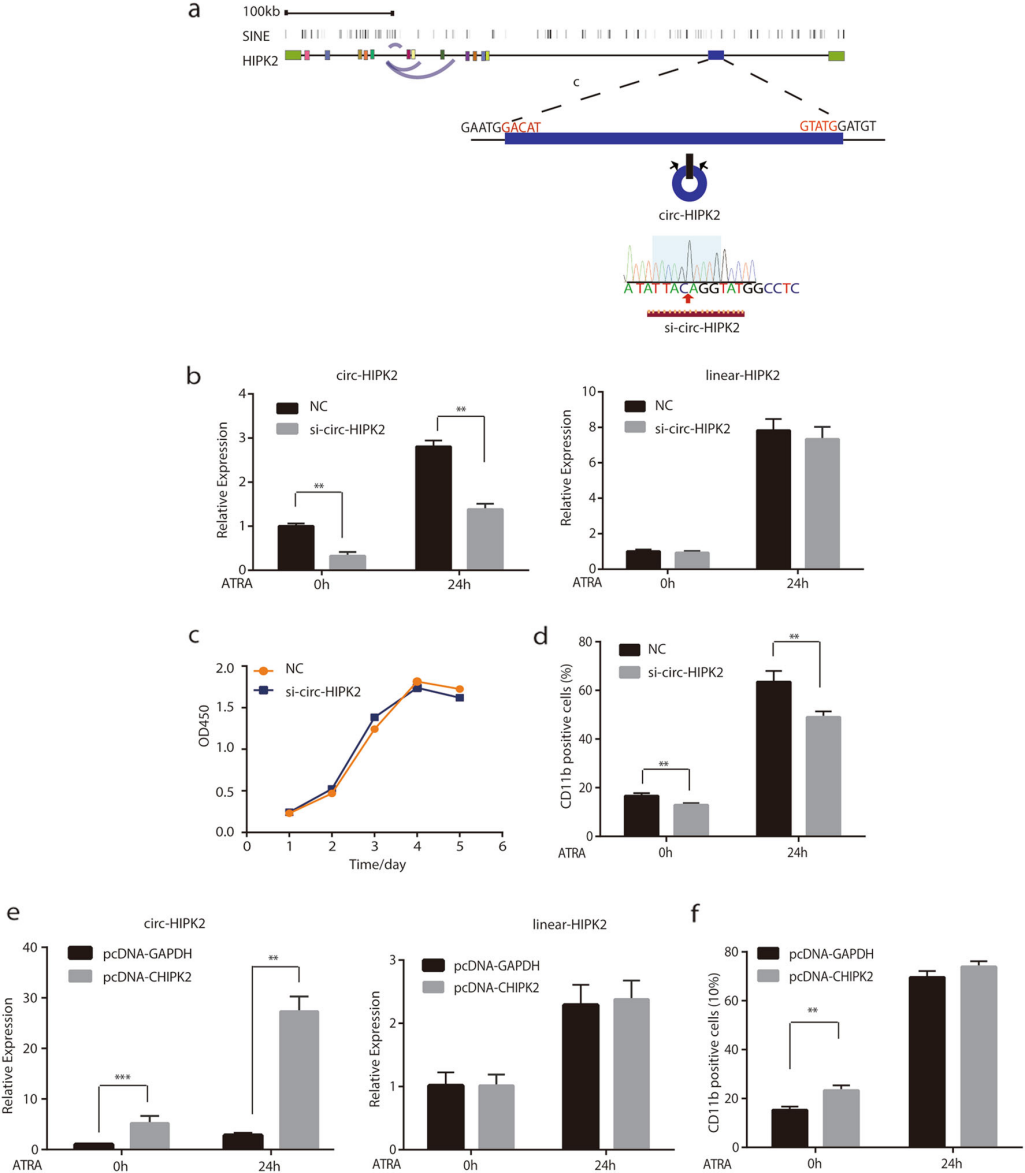
Function of circ-HIPK2 during ATRA-induced differentiation of NB4 cells. **a** Schematic illustration of circ-HIPK2 from the *HIPK2* gene region and siRNA designed according to the junction sequence of circ-HIPK2. **b** qRT-PCR was performed to detect the expression of circ-HIPK2 and linear-HIPK2 in NB4 cells transfected with circ-HIPK2 siRNA or negative control siRNA. **c** Proliferation detection of NB4 cells after transfected with si-circ-HIPK2 at the indicated time. **d** Downregulation of circ-HIPK2 reduced the ATRA-induced differentiation of NB4 cells. **e** The expression of circ-HIPK2 and linear-HIPK2 in NB4 cells transfected with pcDNA-GAPDH or pcDNA-CHIPK2. **f** Overexpression of circ-HIPK2 increased the differentiation ratio of NB4 cells, with less effect in cells treated with ATRA simultaneously. The data are shown with the means ± s. e. m. of three independent biological experiments. ***P < 0.001

To determine the biological functions of circRNAs in the pathogenesis of APL, we selected circ-HIPK2 for the further functional study. Circ-HIPK2 has been reported to participate in the activities of astrocytes and pulmonary fibroblasts^42,43^. *HIPK2*, the host gene of circ-HIPK2, functions as a transcription coactivator in nuclear bodies, and has been reported to be closely associated with the occurrence and development of acute myeloid leukemia (including APL)^37,38^. Circ-HIPK2 was generated from the second exon of the *HIPK2* gene (Fig. 5a). To explore the function of circ-HIPK2, we designed a small-interfering RNA specifically targeting the junction sequence of circ-HIPK2 to reduce its expression (Fig. 5a). As shown in Fig. 5b, the specific siRNA could remarkably reduce the expression of circ-HIPK2 but had no effect on the expression of linear-HIPK2. We examined the effects of circ-HIPK2 knockdown on cell proliferation and cell differentiation, respectively. As shown in Fig. 5c, d, circ-HIPK2 knockdown significantly repressed ATRA-induced differentiation of NB4 cells but had less impact on the proliferation of NB4 cells. On the contrary, the overexpression of circ-HIPK2 increased the differentiation ratio of NB4 cells, although the combined effect of circ-HIPK2 overexpression and ATRA was not that significant (Fig. 5e,f). Therefore, these results indicated that circ-HIPK2 was required for ATRA-induced differentiation of APL cells.

### Circ-HIPK2 acted as a sponge for miR-124-3p

**Fig. 6:**
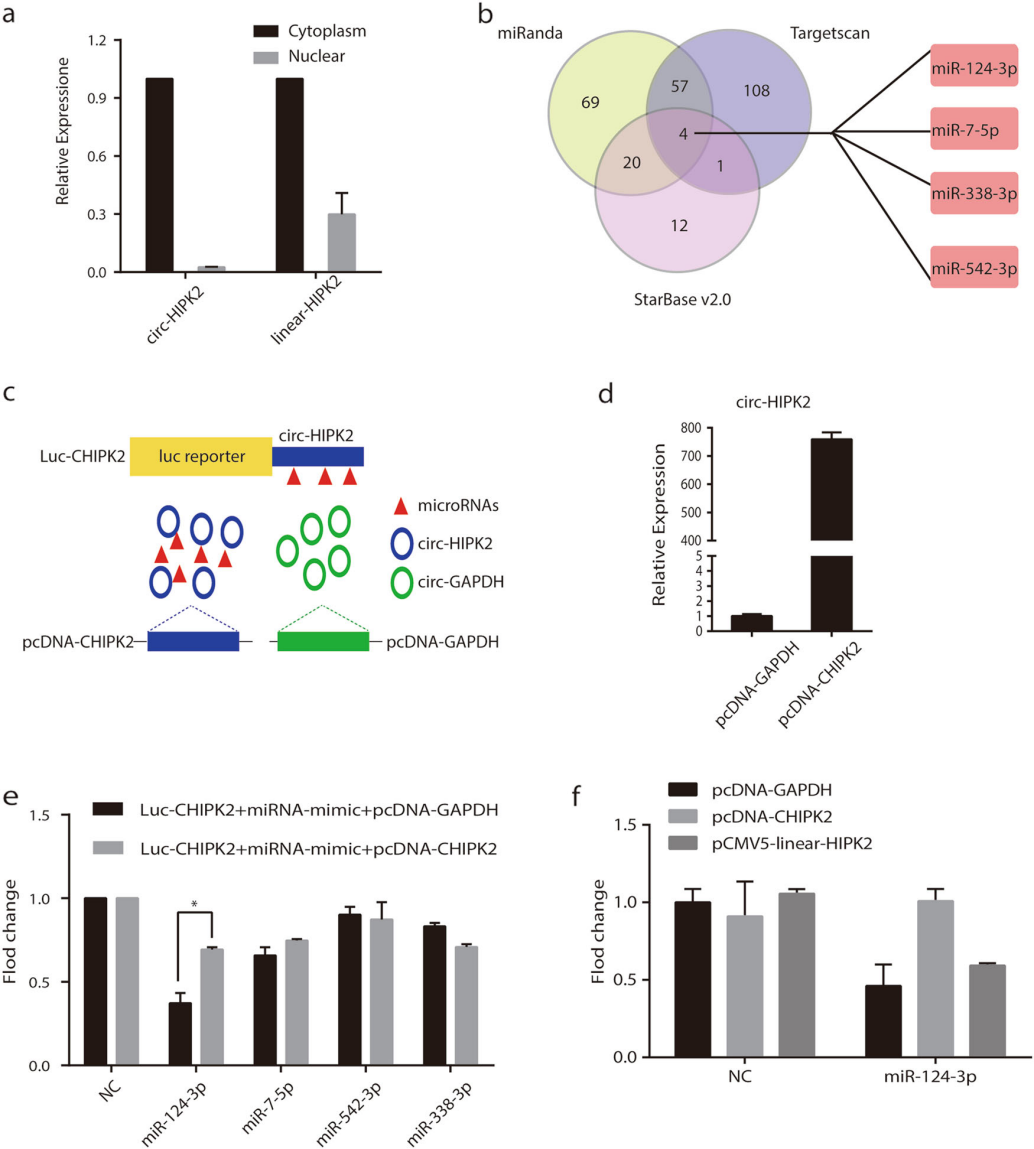
Circ-HIPK2 acted as a sponge for miR-124-3p. **a** The qRT-PCR result shows the relative distribution of circ-HIPK2 and linear HIPK2 in NB4 cells. **b** The target microRNAs of circ-HIPK2 were predicted by three algorithms. **c** Schematic illustration showing the luciferase reporter assay used to screen the target microRNAs sponged by circ-HIPK2. **d** qRT-PCR for detecting the overexpression efficiency of circ-HIPK2. **e** Luciferase activity of Luc-CHIPK2 transfected with microRNA mimics with or without circ-HIPK2 overexpression. **f** Luciferase activity of Luc-CHIPK2 in 293T cells transfected with microRNA mimics when overexpressing circ-HIPK2 or linear-HIPK2. The result is presented with the means ± s. e. m. of three independent biological experiments. *P < 0.05

**Fig. 7:**
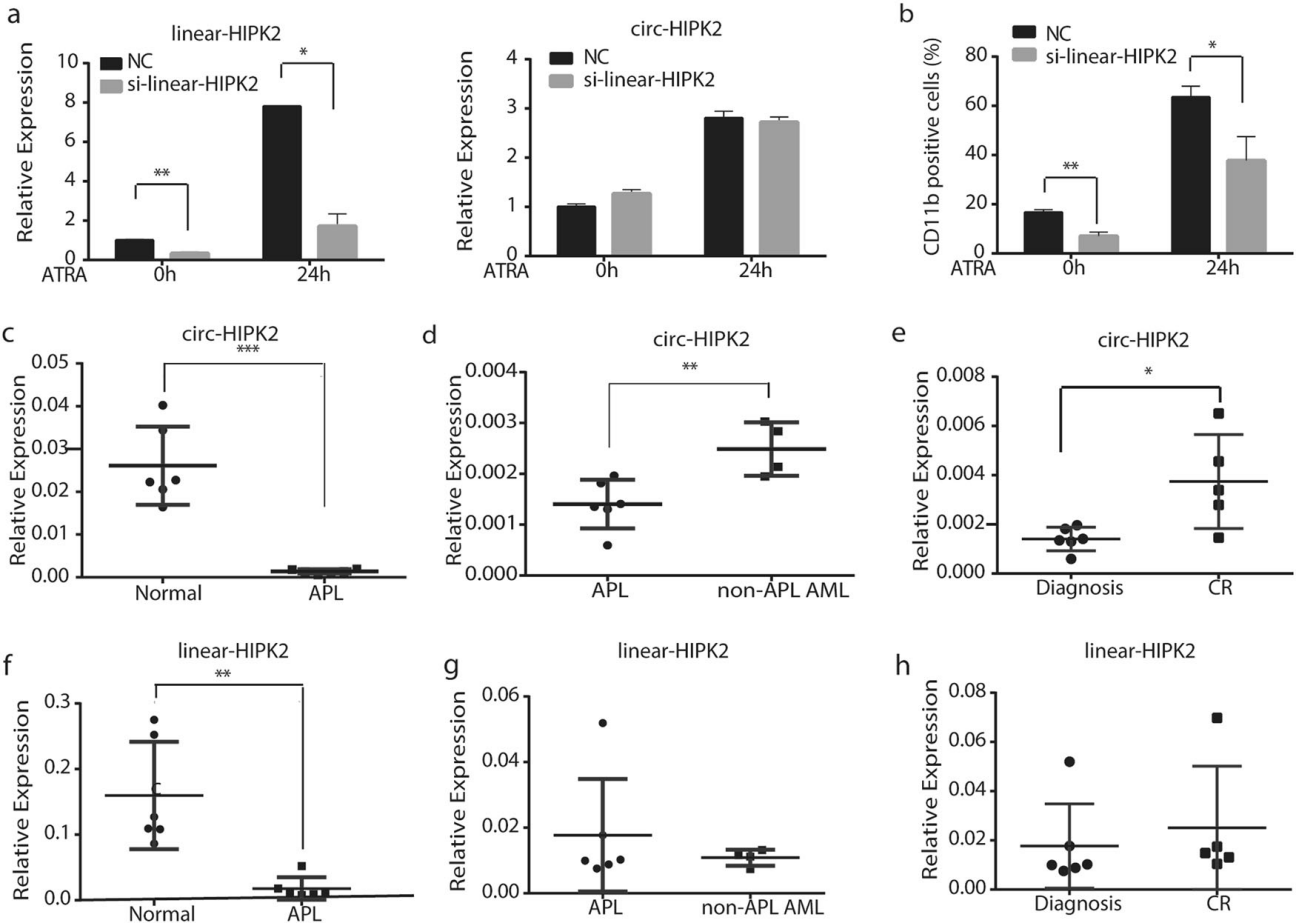
circ-HIPK2 might be a suitable APL biomarker. **a** qRT-PCR was performed to detect the expression of linear-HIPK2 and circ-HIPK2 in NB4 cells transfected with linear-HIPK2 siRNA or negative control siRNA. **b** Downregulation of linear-HIPK2 reduced the ATRA-induced differentiation of NB4 cells. **c**, **d** The expression of circ-HIPK2 in APL patient samples (*n* = 6) was compared with that in normal healthy volunteers (*n* = 6) and non-APL AML patient samples (*n* = 4). **e** The expression of circ-HIPK2 in APL patients at the disease diagnosis (*n* = 6) and at the complete remission state (*n* = 5). CR: complete remission. **f**, **g** The expression of linear-HIPK2 in APL patient samples (*n* = 6) and in normal healthy volunteers (*n* = 6) and non-APL AML patient samples (*n* = 4). **h** The expression of linear-HIPK2 in APL patients at the disease diagnosis (*n* = 6) and at the complete remission state (*n* = 5)

Next, to explore the mechanisms underlying the circ-HIPK2 action in differentiation, we first examined the localization of circ-HIPK2 in NB4 cells. As shown in Fig. 6a, circ-HIPK2 was mainly located in the cytoplasm, while most of linear HIPK2 were distributed in the cytoplasm with a small portion in the nucleus. Since the cytoplasmic circRNAs usually serve as microRNA sponges to regulate gene expression^4^, we hypothesized that circ-HIPK2 might have the ability to act as a competitive endogenous RNA and regulate the level of certain microRNAs in NB4 cells. We used two microRNA prediction algorithms, miRanda^44^ and Targetscan^45^, to search for the potential target microRNAs of circ-HIPK2, and compared them with the results in StarBase v2.0^46^. Accordingly, a total of 4 target microRNAs, i.e. miR-124-3p, miR-7-5p, miR-542-3p, and miR-338-3p, were identified by all three algorithms (Fig. 6b). To verify the candidate microRNAs, we performed a luciferase report assay in HEK-293T cells by inserting the circ-HIPK2 fragment downstream to the luciferase reporter gene (Luc-CHIPK2) and constructing a circ-HIPK2 overexpression plasmid (pcDNA-CHIPK2). The pcDNA-GAPDH overexpression plasmid was used as a negative control (Fig. 6c). As shown in Fig. 6d, the expression of circ-HIPK2 was significantly upregulated in cells transfected with pcDNA-CHIPK2 compared with that in the negative control group. Next, we added four predicted microRNA mimics or negative control mimics into HEK-293T cells with or without overexpression of circ-HIPK2. It was assumed that the candidate microRNAs might repress the luciferase activity if they could bind to the sequence of circ-HIPK2. By detecting luciferase activity, we found that miR-124-3p and miR-7-5p could significantly inhibit luciferase activity compared with negative control. Furthermore, in the cells transfected with pcDNA-CHIPK2, only miR-124-3p could restore the activity of luciferase, showing the competitive ability of circ-HIPK2 to bind to miR-124-3p (Fig. 6e). On the other hand, although down-expression of linear-HIPK2 also impaired the differentiation of NB4, linear-HIPK2 overexpression could not restore the inhibition effect of microRNA-124 on Luc-CHIPK2 reporter gene (Fig. 6f and 7a,b). MiR-124a has the capability to target and reduce the protein level of a key hematopoietic transcription factor, CEBPA^47^. It was deduced that circ-HIPK2 promoted the differentiation of APL cells by serving as a sponge for miR-124-3p.

We finally tested the potential of circ-HIPK2 as an APL biomarker. As shown in Fig. 7c,d, the expression level of circ-HIPK2 was significantly lower in APL patient samples than in normal peripheral mononuclear cells and other subtypes of AML. We also compared circ-HIPK2 expression at the initial stage and complete remission of APL patients. The expression of circ-HIPK2 was significantly elevated when patients were in the stage of complete remission (Fig. 7e). Nevertheless, although linear-HIPK2 also showed down-expression during APL occurrence, it showed no significant change between APL and other subtypes of AML, and between the initial stage and complete remission of APL (Fig.7f–h). These results indicated that circ-HIPK2 might be a suitable APL biomarker.

## Discussion

Exploration of oncogenic regulation in different cancer types has been largely oriented toward protein-coding genes with a recent focus shift to non-coding transcripts. However, little is known about circRNA profiling in leukemia, especially their expression patterns and biological significance. Here we provide the first comprehensive landscape of circRNAs expressed in APL cells and differentially regulated upon ATRA treatment. We performed Ribo-minus RNA-seq (with and without RNase R digestion) using APL patient-derived NB4 cells and identified 4313 APL-expressed circRNAs, of which 1098 were previously unannotated. Furthermore, we uncovered 508 dynamically expressed circRNAs during ATRA treatment, i.e., 246 upregulated and 262 downregulated. This indicated their active involvement of differentiation and the potential function in APL. The present study offers ample scope for future research on circRNA function in APL and ATRA-induced differentiation.

A major achievement of this study is the genome-wide characterization of APL-associated circRNAs, which showed striking features of high abundance, conservation, and cell type-specific expression. In our study, we uncovered a large number of circRNAs with distinct levels of expression, indicating that circRNAs exerted potential function in the initiation and progression of APL. We also showed that the circRNAs expressed in APL could be distinguished from B cells, hematopoietic stem cells and neutrophils, which was consistent with previous findings that circRNAs are expressed in a cell type-specific manner^5,9^, and also suggested that circRNAs may serve a specific function in each cell type. In addition, it should be noted that circRNAs may contribute to the relapse and drug-tolerance of patients due to their unique stability. In fact, the circRNA derived from the fusion site of MLL/AF9 has been shown to confer resistance to arsenic trioxide in acute myeloid leukemia^34^. Furthermore, circRNAs are more likely to accumulate in peripheral blood than linear RNAs^23^. Hence, APL-related circRNAs may act as biomarkers for early diagnosis and prognosis of APL in the future. Actually, the expression level of circ-HIPK2 in APL patient samples was significantly lower than that in normal peripheral mononuclear cells and other subtypes of AML and elevated significantly when APL patients achieved complete remission. More detection and analysis could be done to investigate the potential of circ-HIPK2 as an APL biomarker.

Yet, another important extension of our work would be to elaborate on the internal structure of circRNAs, including the exact exon compositions and alternative splicing events. Several pipelines or algorithms, for example, CIRI-AS^48^, RAISE^49^, and FUCHS^50^, are now available for the high-throughput detection of the internal components of circRNAs. Further, exploration of the internal structures of circRNAs will enrich our understanding on the functional roles of circRNAs in APL.

Notably, we demonstrated that the regulation of most circRNAs were independent of the expression change of corresponding linear RNAs during ATRA-induced differentiation. Coincidentally, during neuron differentiation, the regulation of most circRNAs are also independent of their relative linear RNAs^8^. For example, in glioma, circ-TTBK2 is upregulated and promotes cell growth and invasion, whereas its corresponding linear RNA remains unchanged during this process^51^. On the other hand, a small fraction of regulated circRNAs were in accordance with linear RNAs regulation. In this case, circRNAs might still exert an independent role. For instance, although both circ-ZKSCAN1 and ZKSCAN1 were downregulated in hepatocellular carcinoma cancer, they influenced the proliferation and migration of tumor cells through different biological pathways^52^.

An important question emerging from the profiling observations is whether these APL-related circRNAs have biological functions in APL. In our study, we characterized the expression and function of circ-HIPK2 and revealed that circ-HIPK2 was indispensable for ATRA-induced differentiation of APL cells. The mechanistic study showed that circ-HIPK2 was a sponge for miR-124-3p. Circ-HIPK2 has been reported to function as an endogenous miR-124-2HG sponge to regulate astrocyte activation^42^. Interestingly, miR-124a can target the 3’ untranslated region of CEBPA and reduce its protein level^47^. Thus, circ-HIPK2 might contribute to APL differentiation by sponging miR-124-3p to restore the protein level of CEBPA. More work is needed to determine the molecular mechanisms by which circ-HIPK2 exerts function and to investigate the roles of more circRNAs in APL and ATRA-induced differentiation.

It is worth noting that although RNase R treatment could greatly increase the number of circRNAs detected, it would bring about more cases of false-positives or lead to RNA degradation or cleavage during or after RNA isolation (manifested as not all back-splice events were enriched by RNase R treatment, see also^6,35,53^). Thus, experimental validation is important to identify circRNAs that participate in the pathogenesis and treatment of APL.

In conclusion, we described the first comprehensive landscape of circRNAs expressed in APL cells and their dynamic regulation upon ATRA treatment and demonstrated the important role of circ-HIPK2 during the ATRA-induced differentiation of APL cells. This study provides an abundant resource for future research about circRNAs in APL and brings a new perspective for medicine development and APL-associated biomarker screening.

## Materials and methods

### Human samples

The samples of normal healthy volunteers, APL patients, and non-APL AML patient were obtained from Shanghai Jiao Tong University School of Medicine-affiliated Ruijin Hospital. This study was approved by the ethics committees of Ruijin Hospital and was in accordance with the principle of the Helsinki Declaration II. Written informed consent was obtained from each participant.

### Cell culture and treatment

NB4 cells were cultured in RPMI 1640 (Gibco, Carlsbad, CA, USA) supplemented with 10% fetal bovine serum (Gibco). HEK-293T cells were cultured in Dulbecco’s Modified Eagle’s Medium (Gibco) supplemented with 10% fetal bovine serum. Cells were all maintained in a humidified atmosphere with 5% CO_2_ at 37 °C. All-*trans* retinoic acid (Sigma-Aldrich, St Louis, MO, USA) was dissolved in ethanol as a stock solution at 1 mM. NB4 cells were harvested at 0, 24, and 48 h after the treatment with 1 μM of ATRA.

### Preparation of RNase R-untreated Ribo-minus RNA-seq libraries

Total RNA was isolated using the RNeasy Mini Kit (QIAGEN, Hilden, Germany) according to the manufacturer’s protocol. Three ribo-minus RNA-seq libraries (i.e. untreated NB4 cells, NB4 cells treated with ATRA for 24 h, NB4 cells treated with ATRA for 48 h) were prepared using the TruSeq Stranded Total RNA with Ribo-Zero Globin kit (Illumina, San Diego, CA). The strand-specific libraries were sequenced on the Illumina HiSeq 2500 platform (Illumina, San Diego, CA) with 2 × 125 bp paired-end reads.

### Preparation of RNase R-treated Ribo-minus RNA-seq libraries

Total RNA was isolated using the Hipure Total RNA Mini Kit (Magen) according to the manufacturer’s protocol. Qubit 3.0 Fluorometer (Invitrogen, Carlsbad, CA, USA) and Agilent 2100 Bioanalyzer (Agilent, Santa Clara, CA, USA) were used to analyze the RNA yield and quality. We treated total RNA samples (2 μg) with VAHTSTM Total RNA-seq (H/M/R) Library Prep Kit (Vazyme Biotech) to remove rRNA before constructing RNA-seq libraries. For RNase R treatment, ribosomal RNA-depleted total RNA was incubated for 30 min at 37 °C with 10 units RNase R (Epicentre Technologies, Madison, WI, USA), and then purified with VAHTS RNA Clean Beads. RNase R-treated Ribosomal-depleted RNAs was used for library preparation with the VAHTSTM Total RNA-seq (H/M/R) Library Prep Kit, which included RNA fragmentation and priming, first Strand cDNA Synthesis, second Strand cDNA Synthesis, end Prep of cDNA Library, adaptor ligation, and PCR enrichment of adaptor-ligated DNA. The libraries were then sequenced using a HiSeq X10 (Illumina, San Diego, CA, USA) on a 150 bp paired-end run.

### Data sets

The naive B cells (CD19^+^), hematopoietic stem cells (CD34^+^) and neutrophils RNA-seq data (GSE33772^5^, Illumina GAII, 2 × 80 bp) were downloaded from the Gene Expression Omnibus (GEO).

### Quantification of circular transcripts and linear transcripts and host gene expression

After mapping clean reads to the reference genome with the STAR software^54^, the unmapped reads were used to identify circRNAs according to the principles as previously described^2^. Briefly, the pipeline first focused on the back-spliced junction reads that aligned in the reversed orientation (head-to-tail) as observed in circRNAs. Then, potential circRNAs were further selected if the mapping of mates were consistent with a circular RNA template and the GT-AG canonical splicing sites were presented at circRNA junction borders. Of note, ambiguous breakpoints and candidates from repetitive or homologous regions were all discarded. To estimate the relative expression of a circRNA, we first counted the total number of reads spanning back-spliced junctions (absolute measure of circRNA abundance) and the total number of reads mapped to the human reference genome in each sample, then normalized the number of reads spanning the back-spliced junction to the total number of mapped reads. Genomic coordinates of all detected circRNA candidates were intersected with published gene models (ENSEMBL, release 75).

For the data of RNase R-untreated RNA-seq, the average reads of linear-spliced reads and exon-intron reads close to the back-splice junction at both flanking exons of each circRNA was used as the expression of corresponding linear RNA (as reference^55^ described). Each expression was normalized by dividing the total reads mapped to the whole genome and then multiplied by 100,000,000. TopHat2 and Cufflinks (as described in reference ^56^ were employed to calculate the FPKM value of each host gene.

### Specific and differentially expressed circRNA screening and characteristic analysis

To assess the data reliability of these two sets of RNA-seq data, we calculated the ratio of the overlap circRNAs. On the basis of the pipeline described above, the numbers of circRNA containing at least two junction reads that were identified in RNase R-untreated RNA-seq were 3355 (untreated), 3329 (24-hour post ATRA treatment), and 2721(48-hour post ATRA treatment), respectively. In the RNase R-treated RNA-seq data, the numbers of circRNAs were 14,234 (untreated), 13,715 (24-hour post ATRA treatment), and 9539 (48-hour post ATRA treatment). Approximately 55–60% of the circRNAs identified in the RNase R-untreated RNA-seq data were also detected in the RNase R-treated data.

To identify candidate circRNAs that were APL specific, only circRNAs that were repeated detected in both RNase R-untreated and -treated data sets in at least one of the three time-points (NB4 without ATRA treatment, NB4 treated with ATRA for 24 h, NB4 treated with ATRA for 48 h) were calculated. In total, there were 4313 candidate circRNAs. The following analysis and experiments were all based on these circRNAs.

To explore the differentially expressed circRNAs during ATRA-induced differentiation, we set a 1.5-fold-change as the threshold. We first identified circRNAs that were deregulated in the two timepoints of ATRA treatment. Among the four timepoints [24 and 48 h of RNase R-untreated data, 24 and 48 h of RNase R-treated data], we first selected circRNAs that have at least three time-points with the fold change values greater than 1.5 or less than 0.67 (if three time-points, the fourth timepoint must be greater than 1.0 or less than 1.0). To exclude those that may be false positive, we screened these deregulated circRNAs with the following criterions. Only the circRNAs that have the junction reads ≥3 in either 24 or 48 h timepoints of both RNA-seq data sets could be identified as upregulated circRNA. Similarly, only those circRNAs that have the junction reads ≥3 in the untreated samples of both RNA-seq data sets could be defined as downregulated circRNAs.

To identify the specific circRNAs in each cell type, we compared the circRNAs expressed in NB4, naive B cells (CD19^+^), hematopoietic stem cells (CD34^+^) and neutrophils. Considering the different read length among these RNA-seq data, we first trimmed our RNA-seq reads from 125 bp to 80 bp and re-analyzed the circRNAs using the pipeline described above. The Venn diagram was then employed to select cell-specific circRNAs.

### Validation of circRNAs

Both NB4 cells and patient samples were used to validate circRNAs. RNA was reverse transcribed using SuperScript III Reverse Transcriptase (Invitrogen, Carlsbad, CA, USA). Real-time PCR was performed by Vii7 Real-Time PCR System (Applied Biosystems, Foster City, CA, USA) with the SYBR Green Real-time PCR Master Mix (Takara, Dalian, China). All primers used in this assay are listed in Supplementary Table S5. RNase R (Epicentre, Madison, USA) treatment (3 U/μg) was performed for 30 min at 37 °C.

### siRNA transfection

Small-interfering RNAs (siRNAs) targeting circRNAs were designed at each head-to-tail junction site and synthesized by RiboBio (Guangzhou, China). The specific siRNA sequence for circ-HIPK2 and linear-HIPK2 were as follows: circ-HIPK2, 5′-UUACAGGUAUGGCCUCACATT-3′; linear-HIPK2, 5′-GAGUAAGCAGCACCAGUCATT-3′. A mismatch siRNA sequence was used as negative control: 5′-UUCUCCGAACGUGUCACGUTT-3′. NB4 cells were transfected with siRNAs using Nucleofector Kit V (Lonza, Cologne, Germany) in the Amaxa Nucleofector II device (Lonza, program X-001) according to the manufacturer’s protocol.

### Granulocytic differentiation

CD11b was used to detect the differentiation level of granulocytes. After transfection, harvested NB4 cells were incubated with the anti-CD11b antibody (BD Biosciences, San Jose, CA, USA) for 15 min, and the percentage of CD11b positive cells was analyzed by a Cytomics FC-500 flow cytometer (Beckman Coulter, Miami, FL, USA).

### CCK-8 assay

The proliferation of NB4 cells was tested by the CCK-8 kit (Dojindo, Japan). At 0, 24, 48, 72, and 96 h after transfection, ~1 × 10^4^ transfected cells in 100 μl volume were put in sextuplicate in 96-well plates. Each well was added with 10 μl of the CCK-8 solution and incubated at 37 °C for 4 h. Absorbance was detected using a microplate reader (PowerWave X; BioTek, Winooski, VT, USA) at 450 nm.

### MicroRNAs prediction

To predict the potential microRNAs, two softwares, i.e., miRanda (http://www.microrna.org) and Targetscan (http://www.targetscan.org), were used. The human microRNA library was obtained from http://www.targetscan.org. The micoRNA-circRNA interaction results from CLIP-Seq data were downloaded from StarBase v2.0 (http://starbase.sysu.edu.cn). By comparing the results, the potential target microRNAs predicted by all three algorithms were screened out.

### Plasmid construction

The empty pLuc and pcDNA plasmids used in the luciferase assays were obtained from Prof. Shenglin Huang at Fudan University as previously described^57^. The fragment of the circ-HIPK2 sequence was ligated into pLuc and pcDNA to construct Luc-CHIPK2 and pcDNA-CHIPK2 plasmids, respectively. The linear-HIPK2 overexpression plasmid pCMV-linear-HIPK2 was obtained from Prof. Shengcai Lin at Xiamen University as previously described^58^.

### Luciferase reporter assay

HEK-293T cells were seeded at a density of 1 × 10^4^cells per well in 96-well plates for 24 h before transfection. A total of 50 ng of Luc-CHIPK2, 5 ng of pRL-SV40, 200 ng of pcDNA-CHIPK2 or pcDNA-GAPDH, and microRNA mimics (or negative control) at the indicated concentration were transiently transfected into HEK-293T cells using Lipofectamine 2000 (Invitrogen, Carlsbad, CA, USA). After 24 h of transfection, luciferase activity was measured with a luminometer (Promega) using Dual-Luciferase Reporter Assay System reagents (Promega). The pRL-SV40 plasmid was used as an internal control for transfection efficiency. The negative control microRNA was used for calculating the relative luciferase activity of each target microRNA.

### Statistical analysis

Statistical significant differences were determined by a Student’s *t* test. Data was presented as mean ± s.e.m. *P* values less than 0.05 were considered statistically significant. The data analysis was performed using GraphPad Prism6 (http://www.graphpad.com/) and R software version 3.2.0 (http://www.R-project.org/).

## Electronic supplementary material

Supplementary Materials

Supplementary Figure and Table Legends

Supplementary Figure S1

Supplementary Figure S2

Dataset 1

Dataset2

Dataset 3

Dataset 4

Dataset 5
