# Supplementary material for: Profiling and functional analysis of circular RNAs in acute promyelocytic leukemia and their dynamic regulation during all-trans retinoic acid treatment
Source: Cell Death Dis. 2018 May 29;9(6):651. doi: 10.1038/s41419-018-0699-2 (PMC5973936; doi:10.1038/s41419-018-0699-2)
Supplement: Supplementary file 1 — Supplementary Materials [file 41419_2018_699_MOESM1_ESM.docx]

**Supplementary Figure S1 (TIF 833KB)**

**Supplementary Figure S2 (TIF 1.94MB)**

**Supplementary Table S1 (Excel 13KB)**

**Supplementary Table S2 (Excel 460KB)**

**Supplementary Table S3 (Excel 132KB)**

**Supplementary Table S4 (Excel 67KB)**

**Supplementary Table S5 (Excel 11KB)**
