# Supplementary material for: Profiling and functional analysis of circular RNAs in acute promyelocytic leukemia and their dynamic regulation during all-trans retinoic acid treatment
Source: Cell Death Dis. 2018 May 29;9(6):651. doi: 10.1038/s41419-018-0699-2 (PMC5973936; doi:10.1038/s41419-018-0699-2)
Supplement: Supplementary file 2 — Supplementary Figure and Table Legends [file 41419_2018_699_MOESM2_ESM.docx]

**Supplementary Figure and Table legends**

**Supplementary Figure 1. Validation of circRNAs in NB4 cells.** Another 8 selected circRNAs were confirmed by Sanger sequencing. The back-spliced junction sequence is covered by a blue background and the junction site of each circRNA is indicated by a red arrow.

**Supplementary Figure 2. CircRNAs were differentially regulated during ATRA treatment in NB4 cells.** (a and b) Identification of differentially expressed circRNAs upon ATRA treatment for 24 and 48 hours in the RNase R-untreated RNA-seq data. (c and d) Identification of differentially expressed circRNAs upon ATRA treatment for 24 and 48 hours in the RNase R-treated RNA-seq data. Red points indicate the up-regulated circRNAs, and blue points indicate the down-regulated circRNAs.

**Supplementary Table S1. Data used in this study**

**Supplementary Table S2. CircRNA profiles of NB4 cells before and after ATRA treatment**

**Supplementary Table S3. CircRNA profiles of naive B cells (CD19^+^), hematopoietic stem cells (CD34^+^) and neutrophils**

**Supplementary Table S4. Differentially expressed circRNAs after ATRA treatment in NB4 cells**

**Supplementary Table S5. All primers used in RT-PCR and qRT-PCR experiments**
