# Supplementary figures and images for: Profiling and functional analysis of circular RNAs in acute promyelocytic leukemia and their dynamic regulation during all-trans retinoic acid treatment
Source: Cell Death Dis. 2018 May 29;9(6):651. doi: 10.1038/s41419-018-0699-2 (PMC5973936; doi:10.1038/s41419-018-0699-2)

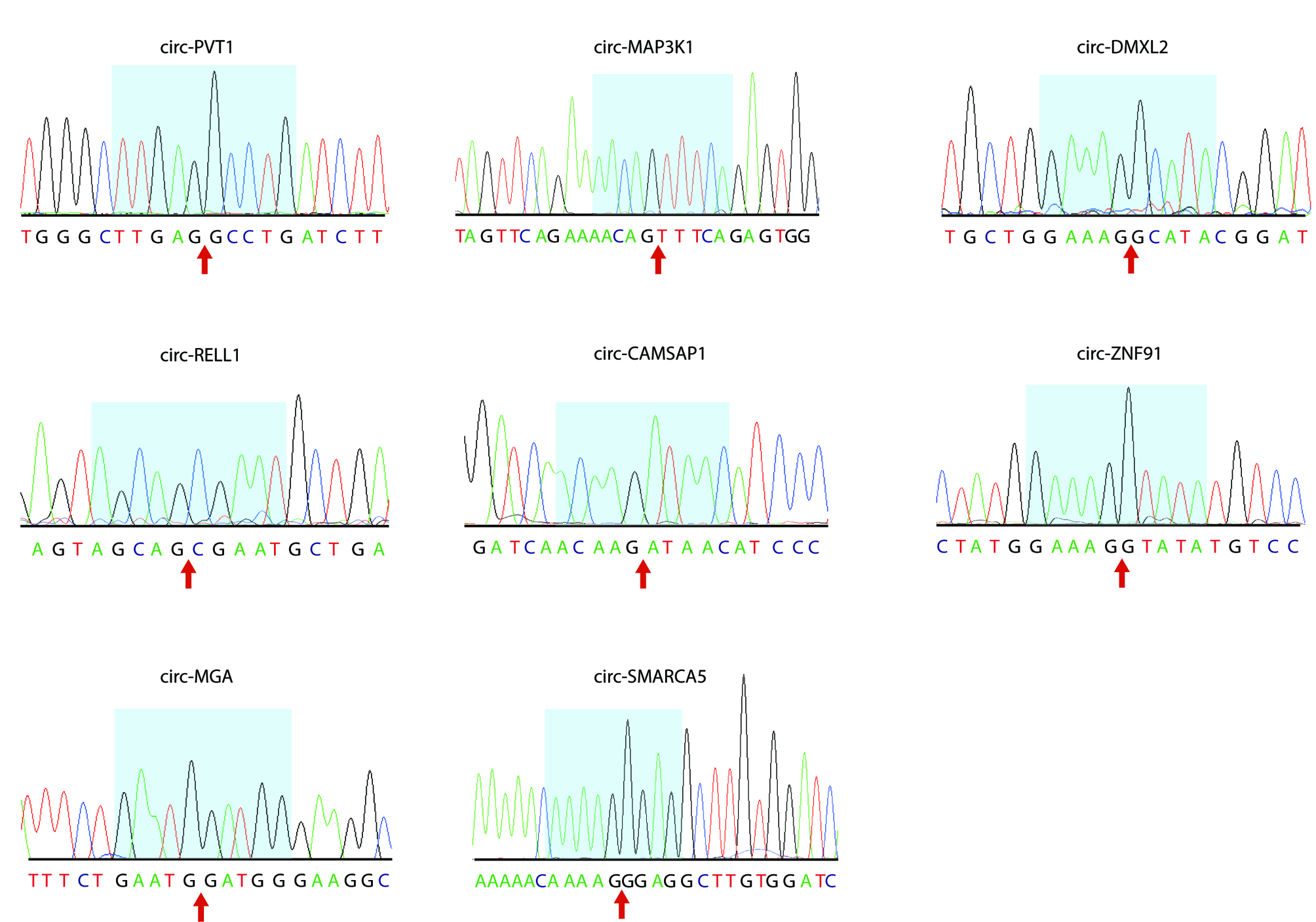

Supplement: Supplementary file 3 — Supplementary Figure S1 [file 41419_2018_699_MOESM3_ESM.tif]

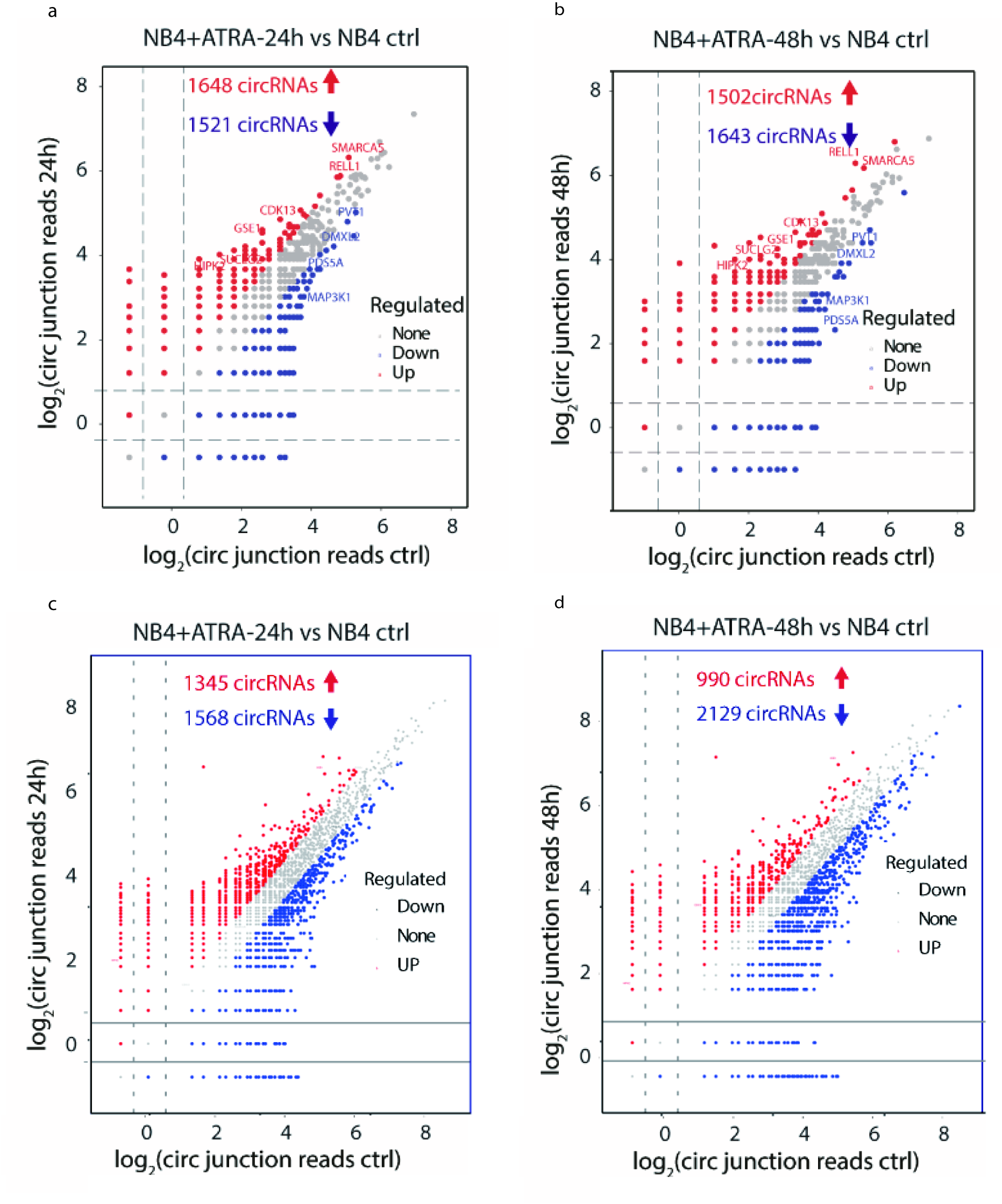

Supplement: Supplementary file 4 — Supplementary Figure S2 [file 41419_2018_699_MOESM4_ESM.tif]
